# Exploring the electrical robustness of conductive textile fasteners for wearable devices in different human motion conditions

**DOI:** 10.1038/s41598-024-56733-8

**Published:** 2024-04-03

**Authors:** Afonso Fortes Ferreira, Helena Alves, Hugo Plácido da Silva, Nuno Marques, Ana Fred

**Affiliations:** 1 Instituto de Engenharia de Sistemas e Computadores-Microsistemas e Nanotecnologias (INESC-MN), Lisbon, Portugal; 2 Instituto Superior Técnico (IST), University of Lisbon, Lisbon, Portugal; 3 Instituto de Telecomunicações (IT), Lisbon, Portugal; 4 Meia Mania Lda, Lousã, Portugal

**Keywords:** Biomedical engineering, Electrical and electronic engineering, Electronic devices, Materials for devices, Sensors and biosensors

## Abstract

Conventional snap fasteners used in clothing are often used as electrical connectors in e-textile and wearable applications for signal transmission due to their wide availability and ease of use. Nonetheless, limited research exists on the validation of these fasteners, regarding the impact of contact-induced high-amplitude artefacts, especially under motion conditions. In this work, three types of fasteners were used as electromechanical connectors, establishing the interface between a regular sock and an acquisition device. The tested fasteners have different shapes and sizes, as well as have different mechanisms of attachment between the plug and receptacle counterparts. Experimental evaluation was performed under static conditions, slow walking, and rope jumping at a high cadence. The tests were also performed with a test mass of 140 g. Magnetic fasteners presented excellent electromechanical robustness under highly dynamic human movement with and without the additional mass. On the other hand, it was demonstrated that the Spring snap buttons (with a spring-based engaging mechanism) presented a sub-optimal performance under high motion and load conditions, followed by the Prong snap fasteners (without spring), which revealed a high susceptibility to artefacts. Overall, this work provides further evidence on the importance and reliability of clothing fasteners as electrical connectors in wearable systems.

## Introduction

Wearable devices have seen wide usage in multiple fields ranging from health monitoring, physical performance assessment, personnel protective equipment and many others^1,2^. While part of wearable devices come in accessory- and rigid-type form factors such as smartwatches and smart rings, the use of wearables containing e-textiles has gained increasing interest, with several advantages to the user. These advantages include increased comfort and ergonomy, which are a result of higher flexibility, stretchability and conformability to the human body shapes^3^. Moreover, e-textile wearables tend to be more lightweight and unobtrusive, giving rise to the concept of ''invisible'' sensor integration, even outside clothing^4,5^. Especially designed to be worn on the body, e-textile wearables present an ideal electronic interface to gather and process human physiological data in real time as well as to improve bodily functions such as blood circulation and wound healing^6–8^. Most often, these electronic elements are either fully embedded^9^ or partially detachable^10^, but the first approach remains highly challenging due to a lack of standards for materials and manufacturing methods, durability, washability and incompatibility of textiles with traditional electronic elements. On the other hand, detachable connections have been successfully integrated in stretchable textiles. For instance, researchers have developed smart garments for electromyography (EMG) monitoring with a textile-board connection employed by laminating a flexible PCB directly on the e-textiles^11^.

Detachable connections have several advantages when compared to embedding approaches. Notably, conductive and sensing textiles are more susceptible to mechanical and chemical wear than the electronic unit, which can be effectively encapsulated. The detachable approach thus allows to reuse the main electronic unit across various e-textile garments over time, even as these garments experience a decline in their sensing or actuating properties. This ensures a more sustainable and cost-effective solution, decoupling the lifespan of the electronic unit from that of the e-textile garment. Therefore, prioritizing a modular design simplifies the e-textile recycling and reuse, improving the overall sustainability of the device as well as the repairability of its electronic components, which has gained a significant importance in society and across multiple industries^12^. Moreover, batteries used in wearables can be sensitive to high temperatures (i.e. upon washing), and its safety concerns can become a development bottleneck due to complex regulatory compliance. This is especially true when batteries are permanently fixed on the garment, although there are ongoing research efforts to storing energy directly on the e-textiles through textile-based supercapacitors^13–15^. On the other hand, wearable devices with a detachable unit can be considered a compromise alternative that has even enabled commercial products. Nonetheless, the rigid electronic interface (e.g. PCB) with the flexible textile relies in connectors that need to be reliable, durable, without negatively impacting the usability and functions of the garment.

Among detachable connectors, snap fasteners are the most used due to their low cost, wide availability, ease of use. Snap fasteners can be used together or alternatively with attachment methods such as sewing, crimping, or soldering. Moreover, these components are compatible with multiple materials, textiles, rigid and flexible PCBs, wires, and offer scalability^16^. Examples of wearable devices employing snap fasteners as detachable connectors include a research-grade garment for blood pressure maintenance in hypertensive patients^10,16^.

Although snap fasteners are mechanically durable, their electrical characteristics are not well known, particularly, the electric durability and electronic artefact footprint. Another potential issue with snap fasteners is the poor adhesion established between the acquisition device and the e-textile garment, as the former can be displaced, tremble, or even fall off under active conditions^17^**.** This leads to inaccurate measurements, which ultimately can lead to incorrect data qualifications or diagnosis^18^. The impact of motion in signal quality is even more acute as there is quantitative evidence of the movement characteristics across body positions, the type of motion, e.g. running athlete vs. walking rehabilitation patient, as well as inter-individual characteristics^18^, and under different environments^19^. Furthermore, even small motion artefacts induce variations that greatly affect sensor data. For instance, in electrocardiogram (ECG) signals, unstable contact between the patient’s skin and the dry electrode due to breathing motion causes changes in the impedance and the resting potential, leading to a distorted ECG baseline. Different research attempts have been made to optimize motion measurements and minimize the effect of sensor positioning, as iterative physical prototyping attempts to optimize and correct sensor positioning depending on the expected motion. This can be laborious due to repeated measurements and prone to error due to the variability in motion execution^20^. Moreover, machine learning methods to estimate scores are evaluated in each iteration, and have also been implemented to mitigate different sources of errors. However, they are limited to a particular configuration of sensors only, require calibrations for each individual, and cannot eliminate device-related errors^19^.

Even though data quality remains one of the most challenging factors in the reliable use of wearable devices, the effect of the connectors in data quality has been widely neglected. In fact, rigid-flexible interconnects in wearable devices have been widely recognized as an unsolved bottleneck^21^. While snap fasteners and magnetic parts have been used as electrical connectors in wearable devices, very limited research, if any, has provided evidence about their electromechanical reliability^22–25^. Most research in which snap fasteners were used in wearable devices have only attributed the source of artefacts to the electrodes-skin interface.

In this work we propose to investigate whether conventional fasteners can be used in e-textile wearables subjected to human movement, namely by assessing the loss of contact in the connecting interface and its impact on continuous and real-time biosignal acquisition. Beyond our immediate interest of biosignal acquisitions for health monitoring, our approach further validates the use of snap fasteners as electrical connectors in a variety of other applications such as interior architecture, the automotive industry and military applications^16,26,27^.

## Results

**Figure 1:**
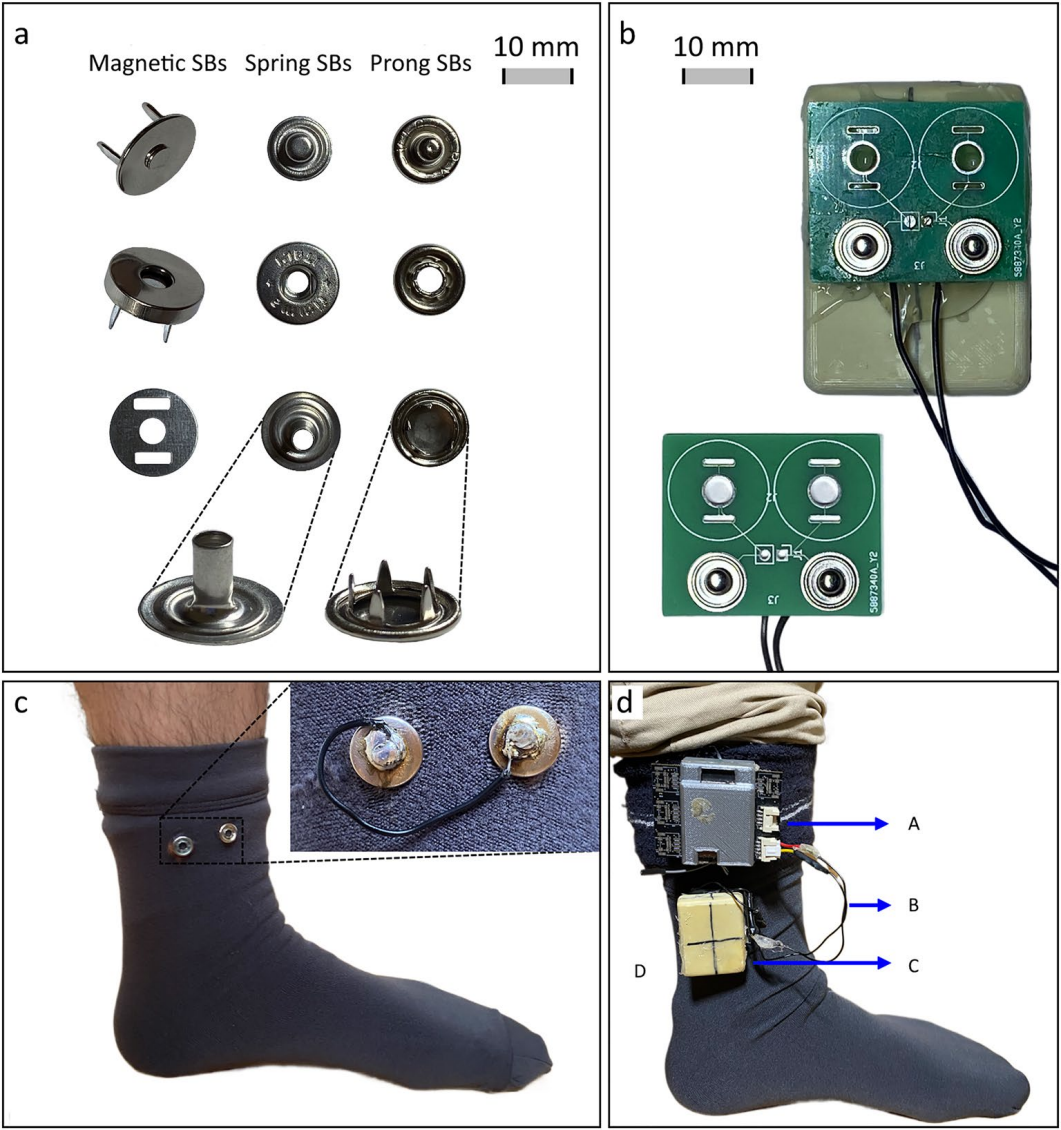
(**a**) Tested fasteners: Magnetic SBs, Spring SBs, and Prong SBs. The bottom parts of the SBs the Spring and Prong SBs are zoomed-in (bottom). (**b**) Interface PCBs with soldered SBs with the test mass (top) and without the test mass (bottom). (**c**) Sock with crimped Prong SBs which were shunted using a wire on the back (upper right; inside out view). (**d**) Test setup with labeled parts: (A) Acquisition Device, (B) Connecting Wires, (C) Interface PCB with test mass, and (D) Sock.

Different snap fasteners, commonly used as electrical connectors in wearable and e-textile applications, were tested for their electromechanical robustness under different motion conditions. Conventional Snap Buttons (SBs) with different sizes, shapes and attachment mechanisms were considered, consisting of the Prong SBs, the Spring SBs, and the Magnetic SBs. While the former two types of SBs are non-magnetic and can be directly soldered to a PCB, in the Magnetic SBs only one of the fasteners' counterparts is magnetic. Thus, for these SBs the non-magnetic one was soldered directly on the PCB, thereby eliminating the issue of losing magnetism due to intense heat applied during the assembly process^23^. Figure 1a shows the three different off-the-shelf fasteners that were tested in this work. In this figure, the back of the buttons are shown for the Prong and Spring SBs, highlighting their different crimping mechanisms.

For the experimental setup, a regular sock was considered as a good form factor for testing since the foot is an anatomical location associated with high motion under realistic human movement conditions (e.g. walking). For each type of tested SBs, a pair of buttons was crimped to a sock. On the inside out of the sock, the pair of SBs was electrically shunted by soldering a wire on their back, as shown in Fig. 1c. It is worth mentioning that an additional regular sock (without fasteners) was worn under the experimental sock to insulate the electrical connections from skin contact and prevent potential electrical conduction through the skin at the crimped SBs. The condition of the wires was also checked during the experiments to ensure reliable connectivity.

To connect the electronic unit to the SBs of the sock, multiple PCBs were customized to which the respective counterparts of the SBs were soldered, as shown in Fig. 1b. To acquire the signals from the garment, the ScientISST CORE was used. This device is an acquisition board especially developed for biomedical applications^28^. The device was also connected to a tri-axial accelerometer and a small battery for power supply, and is showed in the test setup in Fig. 1d (labeled as A in the figure and designated as Acquisition Device). The PCBs were connected to the Acquisition Device through loose wires (eliminating tension) and then attached on the crimped connectors on the sock through the Interface PCB. This approach allowed eliminating the influence of motion of the Acquisition Device on the connection established in the sock. Moreover, it also allowed studying the influence of additional weight on the connectors separately from the Acquisition Device, by gluing a test mass of 140 g to the Interface PCB.

To test the SBs, three movement regimes were considered: static (standing), walking and rope jumping. The acquisitions took 60 s each and were repeated five times for each type of SB and movement regime. Moreover, when performing each of the five repetitions, the interface PCB was connected to the SBs at the sock in alternating positions. For instance, in the first repetition the interface PCB was attached in the default position, and on the second one it was attached upside down, and so forth. This method introduces additional variability stemming from differences in the mechanical fit, which is deemed adequate for the exploratory nature of the experiments. All acquisitions were successfully performed, with no connector mechanically detaching from the sock.

**Figure 2:**
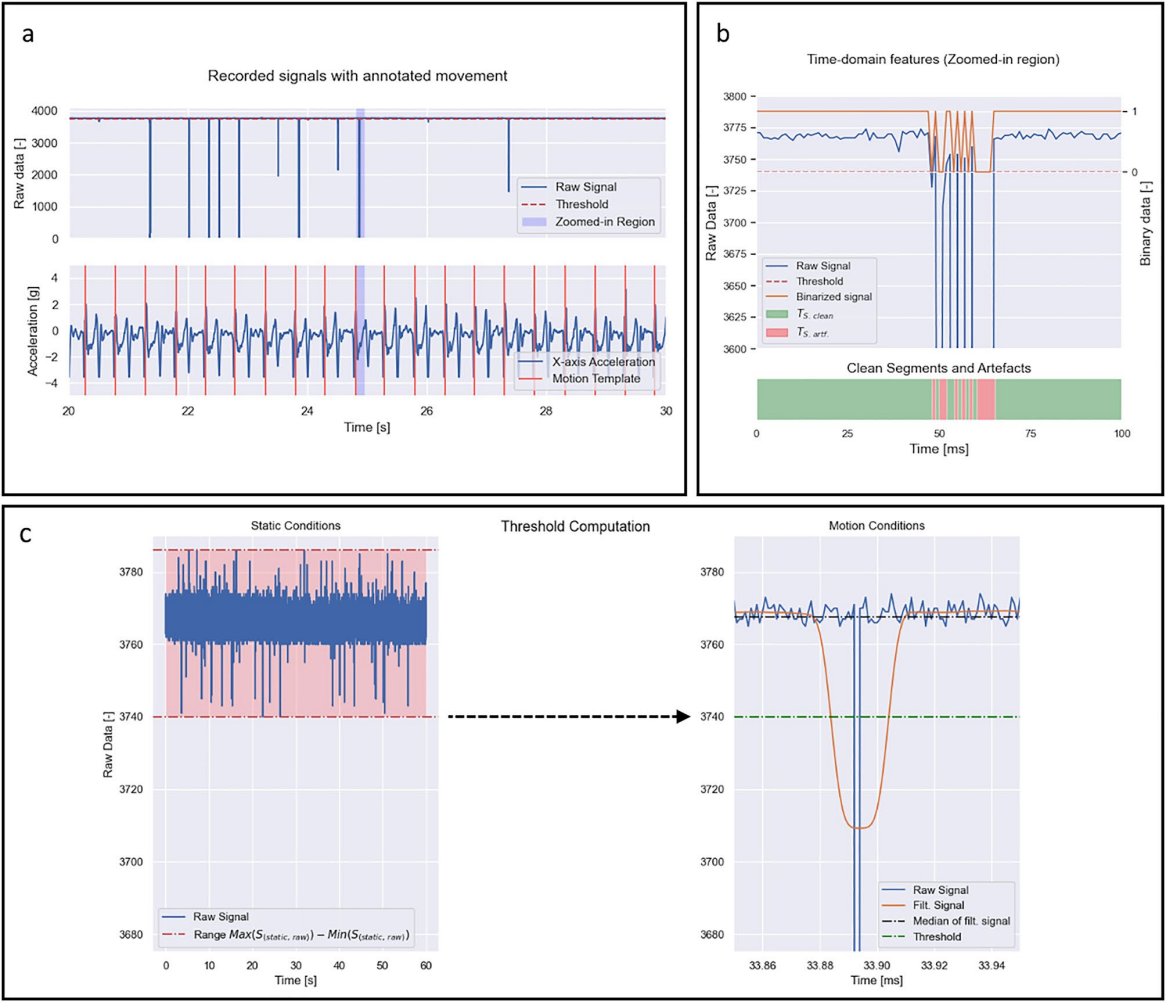
Recorded signals for Prong SBs in Rope Jumping conditions. (**a**) 10-s segment of the analog signal (upper plot), along with X-axis acceleration data with annotated motion events (bottom plot). The sections highlighted in blue refer to the zoomed-in region shown in (**b**). (**b**) Zoomed-in region of the analog signal and its binarized representation (upper plot) and extracted segments of clean signal (green) and artefacts (red) (bottom plot). (**c**) Visual interpretation of the amplitude threshold for artefact detection (left: Prong SBs in static conditions; right: rope-jumping conditions).

Table 1 summarizes the cadence of the movements performed in the walking and rope jumping conditions. As shown, for the first condition, the mean values were approximately 50 steps-per-minute (spm), confirming the correct performance of slow pace/low intensity walking by the subject. Moreover, for rope jumping, the observed ranges did not fall outside 120 jumps-per-minute (jpm), confirming the performance of this movement at moderate-to-high intensity. Figure 2a shows the signal recorded through the connector as well as the annotated motion events for the Prong SBs under walking conditions. To validate that the motion of those conditions was within the expected pace, the durations between consecutive labeled foot landings were computed. During the acquisition, a set of signal artefacts were observed. Figure 2b shows the extracted segments of clean signal and contact artefacts, under the same conditions.

**Table 1:** Statistics obtained from the extracted walking and rope jumping paces in steps-per-minute (spm) and jumps-per-minute (jpm), respectively.

	Walking (spm)		Rope jumping (jpm)	
	PCB	PCB + 140 g	PCB	PCB + 140 g
Prong SBs	50.02 ± 1.95	50.04 ± 1.88	119.53 ± 4.11	119.4 ± 3.96
Spring SBs	49.96 ± 1.52	49.96 ± 1.67	119.87 ± 6.26	119.4 ± 3.64
Magnetic SBs	50.01 ± 1.91	50.0 ± 1.59	119.73 ± 4.5	119.6 ± 4.43

Each value is obtained from the five repetitions and is expressed as mean ± one standard deviation.

The collected signals were analyzed, namely by assessing the frequency and duration of artefacts resulting from loss of contact in the connector. Since the circuit formed by the SBs crimped on the sock was shunt, the most significant artefacts would be expressed by high-amplitude decreases in signal amplitude (i.e. transient loss of contact in the connector). This variation was found to be considerably higher than the baseline variation observed under static conditions, in which case this variation likely resulted from electrical noise (Fig. 2c). Therefore, similarly to previous work, amplitude thresholding was considered to detect the sharp contact-induced artefacts^18–30^. The impact of these artefacts was then assessed under the three different levels of motion.

As a starting point of the signal processing, the Coverage of the Range (${C}_{R}$) was computed, determining the maximum decrease in amplitude observed as a percentage from the maximum value (e.g. 100% when the signal fully saturates at zero). An amplitude threshold was then defined such that it enabled to separate well the high-motion artefacts from baseline amplitude variation, as shown in Fig. 2b). Using this threshold, for motion conditions, the signals were segmented into segments of clean signal (i.e. absence of artefacts) and segments corresponding to artefacts, with time intervals of ${T}_{S clean}$ and ${T}_{S artf.}$, respectively. The maximum duration of ${T}_{S artf.}$ was obtained for each experiment, and the frequency of artefacts ${f}_{artf.}$ [s^−1^] was estimated based on the number of segmented artefacts and acquisition time. Additional metrics were also defined to check the temporal dispersion of artefacts and estimate whether there were enough uncorrupted segments with a sufficient time span (i.e. considering real-time monitoring of biosignal applications). For this, three time intervals were considered, defined as having 1, 2 and 4 s. For each of them, an estimation was computed based on the summed durations of the clean segments, ${T}_{S clean}$, which had durations above that time interval. The estimation was then expressed as a percentage of the total acquisition time. These metrics correspond to ${T}_{d>1 s}$, ${T}_{d>2 s}$ and ${T}_{d>4 s}$, for the time intervals of 1, 2 and 4 s, respectively. These time intervals are on the same order of magnitude of the time period of some biosignals (e.g. for the ECG, the heart-rate at resting conditions is approximately 1 to 1.3 s^31^, and for the respiratory rate it is 5 to 3 s in adults^32^). Therefore, they can be used to test whether it is possible to record one or multiple periods of some biosignal modalities in a continuous and uninterrupted manner.

Table 2 summarizes all the metrics obtained for the different SBs under the various motions. Within all tested connectors, Magnetic SBs stand out, having withstand all conditions without any artefact being recorded, and can be considered optimal. For the Prong and Spring fasteners, under the absence of the test mass, ${C}_{R raw}$ was $<4\%$, the average frequency was lower than 0.02 artefacts s^−1^, and ${Max(T}_{S. artf.})$ was $<1$ millisecond. When the test mass was added, the Prong SBs consistently presented pronounced artefacts and occurring at higher frequencies in motion conditions. For instance, under walking conditions, the artefacts occurred at a frequency of 0.41 ± 0.29 artefacts s^−1^ and covered the measurement range at ${C}_{R raw}=$ 85.34 ± 29.32% (mean and standard deviation). Moreover, there was a high variability for the maximum artefact duration, ${Max(T}_{S. artf.})$, which was 6.6 ± 5.35 ms. For rope jumping conditions, the obtained metrics were similar, except for the artefact frequency which was increased (0.98 ± 0.57 artefacts s^−1^). As for the Spring SBs most metrics did not differ significantly from the control conditions, except for the walking conditions in which ${C}_{R raw}$ 40.64 ± 48.3%. However, the metrics consisting of the artefact frequency and ${Max(T}_{S. artf.})$ were relatively low, with values of 0.02 ± 0.02 artefacts s^−1^ and 0.6 ± 0.49 ms, respectively.

**Table 2:** Metrics extracted from the recorded signals to evaluate the amplitude (${{\text{C}}}_{{\text{R}}}$) and temporal features (${f}_{artf.}$ And ${Max(T}_{S. artf.})$) of the artefacts.

Tested connector	Motion	Mass	${C}_{R} [\mathrm{\%}]$	${f}_{artf. }$[s − 1]	${Max(T}_{S. artf.})$[ms]
Prong SBs	Standing	PCB	1.22 ± 0.04	–	–
	Walking		2.72 ± 2.94	0.0 ± 0.0	0.0 ± 0.0
	Rope jumping		3.84 ± 3.17	0.02 ± 0.03	0.8 ± 1.17
	Standing	PCB + TM	1.24 ± 0.05	0.0 ± 0.0	0.0 ± 0.0
	Walking		85.34 ± 29.32	0.41 ± 0.29	6.6 ± 5.35
	Rope jumping		86.36 ± 27.28	0.98 ± 0.57	6.6 ± 4.36
Spring SBs	Standing	PCB	1.24 ± 0.05	–	–
	Walking		1.26 ± 0.05	0.01 ± 0.01	0.6 ± 0.49
	Rope jumping		1.36 ± 0.19	0.01 ± 0.01	0.6 ± 0.49
	Standing	PCB + TM	1.38 ± 0.21	0.07 ± 0.05	1.0 ± 0.0
	Walking		40.64 ± 48.3	0.02 ± 0.02	0.6 ± 0.49
	Rope jumping		1.36 ± 0.14	0.05 ± 0.04	1.0 ± 0.63
Mag. SBs	Standing	PCB	1.28 ± 0.12	–	–
	Walking		1.28 ± 0.07	0.0 ± 0.0	0.0 ± 0.0
	Rope jumping		1.28 ± 0.04	0.0 ± 0.0	0.0 ± 0.0
	Standing	PCB + TM	1.22 ± 0.04	0.0 ± 0.0	0.0 ± 0.0
	Walking		1.22 ± 0.04	0.0 ± 0.0	0.0 ± 0.0
	Rope jumping		1.22 ± 0.04	0.0 ± 0.0	0.0 ± 0.0

In the column describing the mass of the setup, TM stands for Test Mass. Each value is obtained from the five repetitions and is expressed as mean ± one standard deviation. The metrics ${f}_{artf.}$ And ${Max(T}_{S. artf.})$) are not shown for the control conditions (standing static without the added test mass) since the threshold was derived from the acquisitions performed under these conditions.

**Figure 3:**
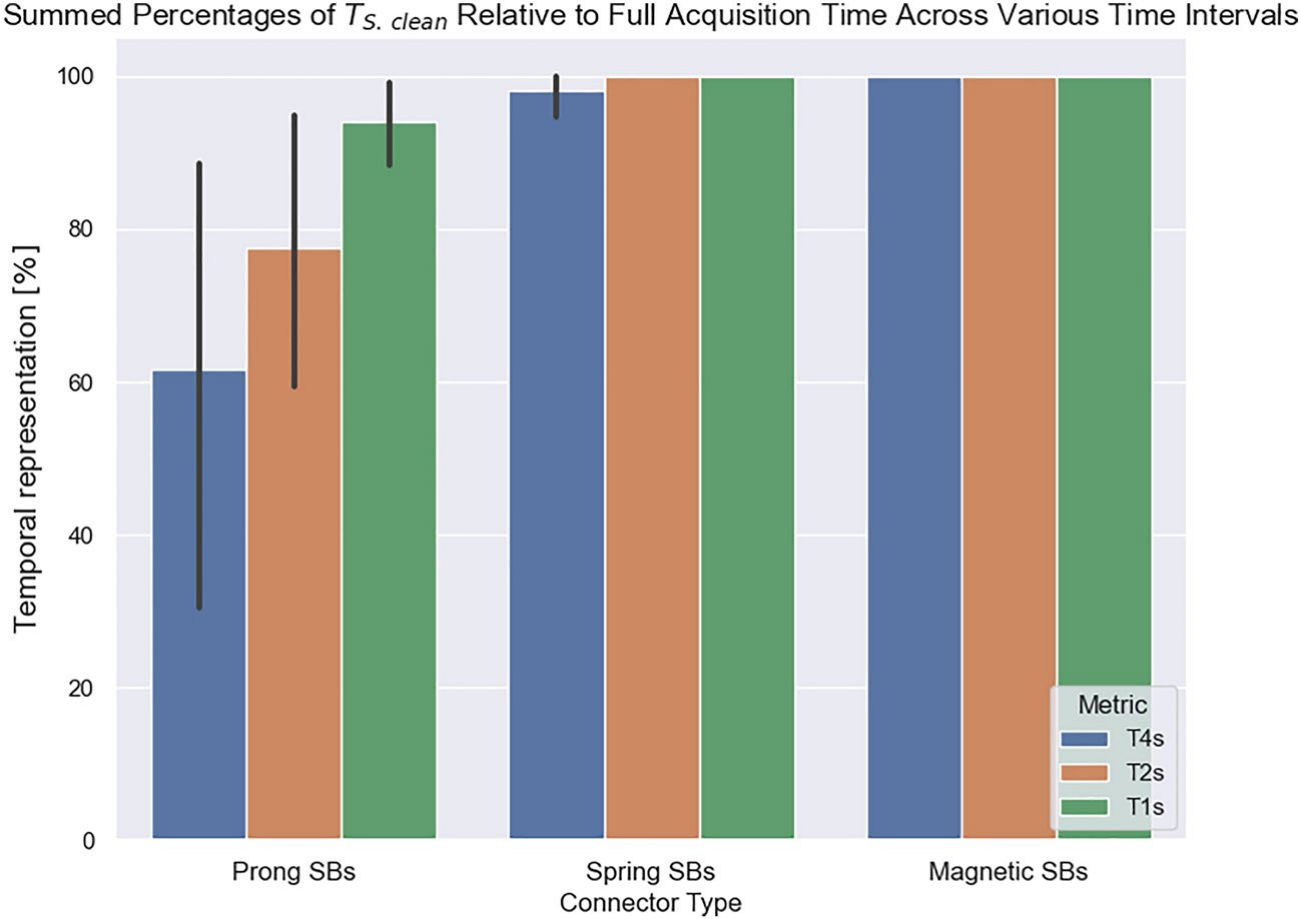
Total duration of clean signals as a percentage of the total acquisition time for the three time intervals given by ${T}_{d>1 s}$, ${T}_{d>2 s}$ and ${T}_{d>4 s}$. Each bar represents the mean value with 95% confidence intervals obtained from the five repeated measurements.

To further study the effects of the artefacts on these fasteners and consider their statistical variability, an analysis of the time intervals of clean signals under the different motions was also performed. In Fig. 3 the percentage of clean segments (${T}_{S. clean}$) was plotted for all the connectors under walking conditions with the added test mass. As shown, for the Prong SBs, the acquired signals are not fully represented by artefact-free segments. For instance, considering the ${T}_{d>4 s}$ metric, segments of clean signal with a duration of > 4 s represented approximately between 40 to 90% of the collected signals, whereas this metric was approximately 100% for the Spring SBs, and 100% for the Magnetic SBs. For the metrics ${T}_{d>2 s}$ and ${T}_{d>1 s}$ the values increased but were still sub-optimal. This confirms that the dispersion of artefacts obtained from the Prong SBs led to a limited number of artefact-free segments extracted from the signal.

## Discussion

When using an interface PCB without the added mass all fasteners performed similarly well under all motion conditions. For these conditions, the obtained values for ${C}_{R raw}$ was < 4%, suggesting the registered artefacts did not extend significantly in amplitude beyond the amplitude threshold defined for artefact detection. Moreover, the artefact frequencies were < 0.02 artefacts s^−1^ and ${Max(T}_{S. artf.})$ was under 1 ms (i.e. artefacts were short in duration and occurred in a few number). In some applications, the impact of the artefacts obtained under these conditions on signal acquisition can be considered minimal. For example, for biosignal modalities such as EDA and body temperature, the artefacts can be filtered out using a moving average filter. Considering a 1-ms artefact with an amplitude decrease of 4%, it can be estimated that a moving average filter with a window size of 200 ms would smooth the artefact resulting in an amplitude variation below the ADC quantization error (considering the acquisition setup used in this work; 12-bit ADC acquiring at 1 kHz). Moreover, given the typical periodicity of some biosignals, a segmentation approach would still allow preserving a high number of artefact-free waveforms. For instance, on average, in a 60-bpm ECG signal only one in sixty templates would be contaminated with an artefact.

As for the experiments performed with the additional test mass, the artefacts registered under static conditions did not differ significantly in amplitude from the control. However, this was not the case for the Prong SBs tested under motion conditions. Compared to the control conditions, the artefacts had higher amplitude, duration and frequency. Under walking conditions, the artefact frequency was 0.41 ± 0.29 artefacts s^−1^ (i.e. on average, occurring every 2.44 s), and the measurement range was covered at 85.34 ± 29.32% (mean and standard deviation), which was much higher compared to static conditions. Moreover, the obtained artefact duration was 6.6 ± 5.35 ms. For rope jumping conditions, the obtained metrics were similar, except for the artefact frequency which was higher (0.98 ± 0.57 artefacts s^−1^). For these cases, moving average filters may not be used effectively for artefact removal in post-processing. On the other hand, due to the high amplitude and step-like waveform of the artefacts, the signals may be highly susceptible to ringing effects when using other filtering techniques that are often used in biosignal applications^33^ (e.g. IIR filters). Based on the dispersion of extracted segments, the Prong SBs also demonstrated limited usability for monitoring typical biosignals in artefact-free conditions, especially for signals with frequencies < 1 Hz. For instance, considering the typical heart rate of adults of 60 beats-per-minute (bpm)^31^, a limited number of uncorrupted consecutive ECG templates could be extracted based on the obtained metrics. While snap fasteners have been validated for transmission of high-speed digital data in e-textiles in steady applications^26,27^, this work suggests that Prong-type snap fasteners should be used with caution in wearable applications subjected to high levels of motion and load.

As for the Spring SBs, the observed artefacts were more significant for walking versus rope jumping conditions with the added test mass. In walking conditions, the mean value for ${C}_{R}$ was 40.64%, but the mean values for artefact duration and frequency did not, however, differ from static conditions. Moreover, the dispersion of extracted segments demonstrated that artefact-free segments still almost fully represented the total acquisition time. Considering the aforementioned metrics, it can be concluded that a few number of pronounced artefacts have occurred at walking conditions by chance but not in rope jumping conditions. Overall, the superior performance of this fastener compared to the Prong SBs may result from the spring-based mechanism that engages the plug (male stud). This mechanism possibly increases contact under motion conditions, despite the observation of some high amplitude artefacts.

The Magnetic SBs had an excellent performance as no artefact was registered for any tested condition. Therefore, under high motion and load, this type of connectors are expected to preserve high electromechanical stability. The ability to maintain constant physical contact between the two counterparts is likely attributed to a combination of factors, including the strong magnetic force, flat surface, and large surface area of the coupled Magnetic SBs. These fasteners can thus be considered a reliable connector once the connection is established, with additional benefits such as being more user-friendly compared to other types of fasteners in biosignals sensing^34,35^. It is nonetheless worth mentioning that due to the magnetic properties, current induction and its potential impact on the quality of the acquired signals cannot be ruled out without applications-specific tests (i.e. the signal modality being measured).

Overall, in scenarios characterized by low movement, all types of SBs demonstrate suitability for a health monitoring using a wide range biosignal modalities, with minimal artefacts anticipated. Thus, in applications devoid of high-motion conditions, such as health monitoring within sedentary populations, the selection criteria for SBs should consider the cost, weight, and size of the SBs, the ease of attachment and detachment of the electronic unit from the garment, and the impact of magnetism on both the electronic unit and surrounding e-textiles. Conversely, for applications involving significant motion and load, Spring and Magnetic SBs are preferred. These applications span a variety of domains, including fitness tracking across diverse sports, personal protective equipment, and medical devices, contingent upon specific use cases. Notably, within the latter two fields, ensuring minimal susceptibility to noise and motion artefacts is more significant due to stringent regulatory constraints, in contrast to wearables targeting general consumer markets.

One of the limitations of this study is that the obtained findings may not generalize to scenarios with different levels of human movement and / or load, as the influence of weight can also depend on other factors such as the type (i.e. rotation and translation) and complexity of the movement, as well as the body location and form-factor of the garment. Regarding the connection established by tested fasteners, this study does not consider the possible slippage of the loaded unit, which can also play a role on the impact of the load on the connection established at the garment. Future work should focus on the evaluation of replicate snap fasteners and the replication of permanent attachments at the garment and interface PCB, which may introduce some variability.

## Conclusions

In conclusion, the presented approach explores how connectors can influence sensors based on human movement analysis and wearable system design. With the motion sensor data effects, this methodology enables wearable designers and algorithm developers to analyze and evaluate the impact of connectors and use the best suited regarding motion, wearability and robustness. The presented work provides foundations to design wearable systems subjected to lower movement-induced artefacts, hence maximizing opportunities for biosensor performance optimization. Open questions remain, namely regarding the endurance of the fasteners under repetitive mating/de-mating cycles and the influence of washing cycles on the reliability of the connection. Moreover, although the use of snap fasteners in clothes do not tend to pose significant risks to the human health, it is important to consider the biocompatibility of the metals and/or metallic coatings of clothing fasteners, which may vary depending on these materials, the specific use-case and the form-factor.

## Methods

### Fabrication of the test samples

**Figure 4:**
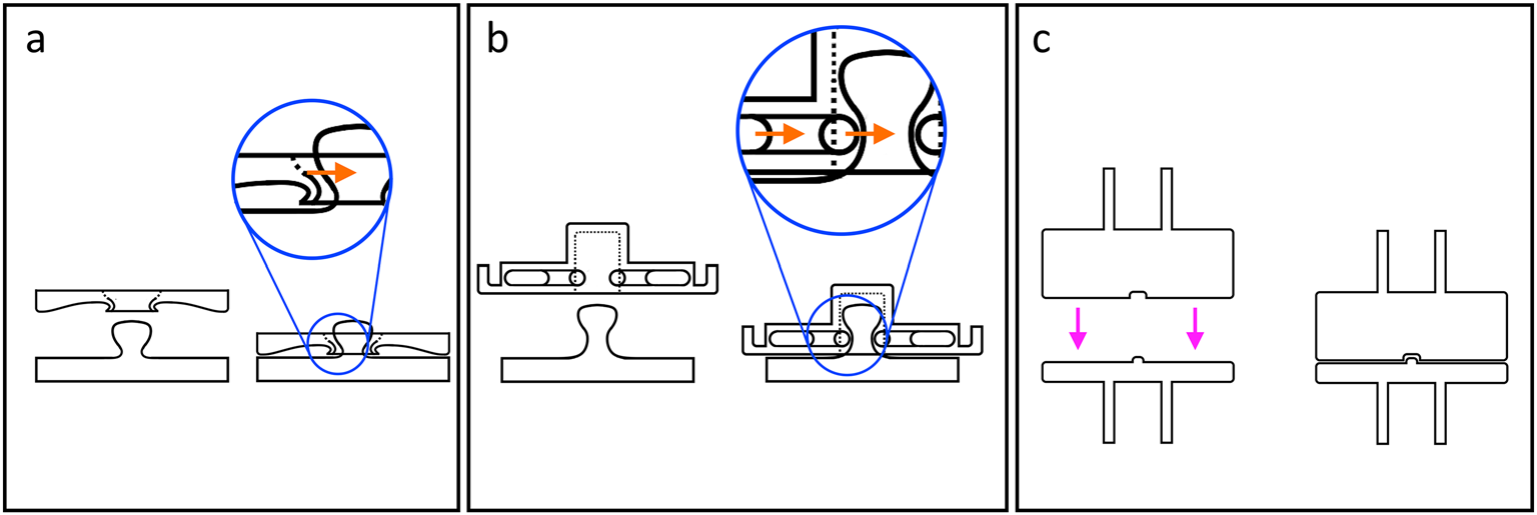
Illustration of the different attachment mechanisms for each of the three tested fasteners. The SBs are represented by their cross-sections. In each subfigure, the SBs shown on the left are detached and the SBs shown on the right are attached. (**a**) Prong SBs, with a zoomed-in view highlighting the force (orange arrow) exerted by the receptacle (top part) laterally on the plug part (male stud, at the bottom). The receptacle part deforms slightly to achieve a press fit. (**b**) Spring SBs, with a zoomed-in view highlighting the force exerted by the spring within the snap fastener acting upon the plug (orange arrows). A press fit is achieved through the compressive spring. (**c**) Magnetic SBs, highlighting the magnetic force exerted by the upper part (pink arrows). The mechanical fit is maintained by the magnetic force and guided by a depression and a protrusion in the middle in the magnetic (top) and the non-magnetic parts (bottom), respectively.

All tested buttons were generic, commercially available unbranded buttons used in clothing. While the Prong SBs crimp to the fabric through a circular array of teeth, the medium SBs do so through a hollow cylinder that deforms outwards (in clothing industry, these buttons are known as Prong and Spring SBs, respectively). As for the Magnetic SBs, they are crimped manually by bending it against a small metallic disc. Regarding the connection between matching SBs, the Prong and Spring SBs achieve mechanical contact through a press fit. As for the Magnetic SBs, the attachment is achieved and maintained based on the magnetic force. Figure 4 details the attachment mechanisms of the three types of SBs.

Polyamide-based socks were used, to which the different test connectors were crimped. After crimping, a metallic wire was soldered to the back of the buttons, electrically shunting them. It is worth mentioning that the wire was left loose to avoid pulling or detaching while performing the experiments. On the acquisition device, the buttons were soldered to customized PCB serving as the interface between the device and the sock (Interface PCB). The device used for the test signal acquisitions contained the ScientISST CORE^28^, a tri-axial accelerometer (PLUX) and a small battery for power supply. The ScientISST CORE has an integrated Bluetooth module for wireless transmission of the recorded signals. It has a controllable sampling rate, ${f}_{s}$, although 1 kHz was used in the experiments in this work. Moreover, an ADC (Analog-to-Digital Converter) resolution of 12-bit^28^ was considered.

### Experimental protocol

The protocol consisted in performing the signal acquisitions under different motion conditions for each connector being tested. For each condition, the acquisition took 60 s and was repeated five times. Before each acquisition, the interface PCB was detached and re-attached to the sock at the connectors, alternating its relative position (e.g. default position, then upside down, and so forth).

The first condition was at rest while standing (Standing Static), consisting of the control setting regarding movement. Then, the acquisitions were performed while walking at slow pace (< 100 steps-per-minute^36^) followed by performing rope jumping at a moderate-to-fast pace (120 jumps-per-minute). Taking into account the diversity of wearable device form-factors, the experiments performed in the aforementioned movement conditions were repeated without additional mass (i.e. the mass of the connector, considered negligible) and then with the additional mass of 140 g. This accounts for a wide range of weights by incorporating both best and worst-case scenarios, respectively, providing insights into connector performance under varying practical conditions. The analog signals recorded when testing the connector, along with the tri-axial acceleration signals, were stored for analysis. The acceleration signal corresponding to the X-axis (i.e. with the same orientation and direction as the blue arrows in Fig. 1d was used to visualize movement and manually label foot landings for walking and rope jumping conditions.

All methods described in this study were conducted in strict accordance with the applicable guidelines and regulations. The experimental protocol employed in this study was approved by the Ethics Committee of Instituto Superior Técnico, University of Lisbon (ethical opinion Ref. no. 9/2022 “Feasibility of e-Textiles for Physiological Measurements at the Foot” issued on 7 June 2022). Informed consent was obtained from the subject involved in this study prior to their participation.

### Data processing

The Coverage of the Range, ${C}_{R}$, given by the ratio of the amplitude range of the raw signal $S$ over its maximum observed value, is given by Equation (1).

1 $$\begin{array}{c}{C}_{R}=\frac{Max\left(S\right)-Min(S)}{Max(S)} .\end{array}$$

As for the amplitude threshold, an amplitude value was defined for distinguishing high-amplitude contact artefacts from the baseline variation from the e-textile. For the purpose, the amplitude variation observed under the static condition was considered (i.e. standing static with no additional mass; no contact artefact was ever found for any connector in those conditions). The threshold was then obtained from the minimum amplitude value found for the five acquisitions under these conditions. The formula for computing the threshold is shown in Equation.

2 $$\begin{array}{c}Th.={min}_{i=1}^{5}(Min\left({S}_{static_{i}}\right) )\end{array}$$

To segment the signal into segments of artefacts and segments of clean signal, a binarized representation of the signal was used by assigning its values to 1 when $S>$ Th. and 0 otherwise.

To estimate the frequency of the artefacts, ${f}_{artf.}$ [s^−1^], the number of artefacts was divided by the length of the acquisition time. To estimate the durations of the two types of segments (clean signal and artefacts), the time instances were identified in which the binarized signal transitioned between 1 and 0 (and vice-versa) and extracted the time durations between consecutive transitions. The time durations of segments of clean signal, ${T}_{S clean}$ were extracted from portions binarized into 1, as well as time durations of segments corresponding to artefacts, ${T}_{S artf.}$, from portions binarized into 0. To rule out the possibility that artefacts were temporally concentrated (i.e. occurring “all at once”) and, therefore, the most part of the signal was unaffected, three additional metrics were computed. These metrics, ${T}_{d>1 s}$, ${T}_{d>2 s}$ and ${T}_{d>4 s}$, quantified the total duration of segments of clean signal with a duration longer than 1, 2 and 4 s, respectively, expressed as a percentage of the overall acquisition time.

## Data Availability

The datasets generated during and/or analyzed in the present study are available from the corresponding authors on reasonable request.
